# Challenges of acute peritoneal dialysis in extremely-low-birth-weight infants: a retrospective cohort study

**DOI:** 10.1186/s12882-020-02092-1

**Published:** 2020-10-19

**Authors:** Jihyun Noh, Chae Young Kim, Euiseok Jung, Joo Hoon Lee, Young-Seo Park, Byong Sop Lee, Ellen Ai-Rhan Kim, Ki-Soo Kim

**Affiliations:** 1 Department of Pediatrics, University of Ulsan College of Medicine, Asan Medical Center, 88, Olympic-ro 43-gil, Songpa-gu, Seoul, 05505 South Korea; 2 Department of Pediatrics, Kyung Hee University School of Medicine, Seoul, South Korea

**Keywords:** Acute kidney injury, Infants, Extremely low birth weight, Peritoneal dialysis, Hyperkalemia

## Abstract

**Background:**

Peritoneal dialysis (PD) has been used occasionally in extremely-low-birth-weight (ELBW) infants with acute kidney injury (AKI). This study aimed to evaluate the clinical characteristics and outcomes of ELBW infants with AKI treated with PD.

**Methods:**

In this retrospective cohort study, the medical records of ELBW infants with AKI, who underwent PD from January 2008 to February 2018, were reviewed. A PD catheter (7.5–9.0 Fr) or central venous catheter (4 Fr) was used for the peritoneal access. Treatment with PD solutions (2.5 or 4.25%) was started at 10 mL/kg, which was increased to 20–30 mL/kg for 60–120 min/cycle continuing for 24 h.

**Results:**

Twelve ELBW infants (seven male and five female infants) were treated, and their mean (±SD) gestational age and birth weight were 27.2 (±3.3) weeks and 706.5 (±220.5) g, respectively. Two patients had severe perinatal asphyxia (5-min Apgar score ≤ 3). The most important indication for starting PD was AKI due to sepsis. The average (±SD) duration of PD was 9.4 (± 7.7) days. The potassium levels in the ELBW infants with hyperkalemia decreased from 6.8 to 5.0 mg/mL after 9.3 (± 4.4) days. The most common complication of PD was mechanical dysfunction of the catheters, such as dialysate leakage (75%). Two patients were successful weaned off PD. The mortality rate of the infants treated with PD was 91.7%.

**Conclusions:**

In this series, the mortality rate of ELBW infants with AKI treated with PD was relatively high because of their incompletely developed organ systems. Therefore, the use of PD should be carefully considered for the treatment of ELBW infants with AKI in terms of decisions regarding resuscitation.

## Background

Although the precise incidence and prevalence rates of acute kidney injury (AKI) among newborns are unknown, it is commonly observed in the neonatal intensive care unit (NICU), with rates approximately ranging from 8 to 24% [1]. Neonates with AKI have very high mortality rates, i.e., 4.5–78% [1–3]. AKI has a significant impact on the survival rates of preterm infants. Because the kidneys of extremely-low-birth-weight (ELBW; birth weight, < 1000 g) infants are immature and susceptible to environmental factors, including infectious pathogens and nephrotoxic medications, prevalence of AKI among ELBW is around 56% [4, 5]. Renal hypoperfusion and ischemia, with or without septic shock, account for 18% of AKI among ELBW infants [4, 6, 7].

The treatment of the metabolic complications of AKI includes appropriate management of fluids, electrolytes, and acid-base balance and appropriate nutrition [8, 9]. Despite the non-dialytic management of AKI among newborns, the major indications for initiating renal replacement therapy (RRT) include severe oliguria despite fluid therapy, diuretic administration, and inotropic support; refractory electrolyte imbalance; and worsening uremia [8–10]. Peritoneal dialysis (PD) is the most common form of RRT in young children, including neonates, because of the relative ease of access and technical simplicity [10–12]. A few studies reported that PD was effective for the management of AKI and metabolic disturbances in neonates, including preterm infants [10, 13]. However, it has been used only occasionally in ELBW infants with AKI because of unavailability of small-sized catheters and volume cyclers [14]. Only a few studies have investigated the implementation of PD in ELBW infants. Therefore, we aimed to evaluate the clinical characteristics and prognosis of ELBW infants who received PD treatment for AKI.

## Methods

### Study population

This study was a retrospective cohort study of ELBW infants born at Asan Medical Center Children’s Hospital, a tertiary academic center, and admitted to the NICU between January 2008 and February 2018. Among them, 12 ELBW infants (seven male and five female infants) required PD for AKI. We defined AKI as any of the following criteria using the modified Kidney Disease - Improving Global Outcomes (KDIGO) definition: increase in the serum creatinine (Cr) level by ≥0.3 mg/dL (≥ 26.5 μmol/L) within 48 h; increase in the serum Cr level by ≥1.5 times the trough level, which was known or presumed to have occurred within the prior 7 days; or urine volume of < 0.5 mL/kg/h for 6 h [15].

### Indications of dialysis

We applied PD in the ELBW infants with AKI, including anuria since birth or oliguria lasting over 48 h; fluid overload, including pulmonary edema; refractory hyperkalemia; severe metabolic acidosis; and uremia. Further, we considered the overall conditions of the patients before and after dialysis and the prognosis in relation to the underlying diseases. Their complications and outcomes were evaluated on the basis of their clinical manifestations and results of biochemistry and serology studies, simple X-ray, and echocardiography.

### Dialysis technique

In all patients, we inserted the catheter for PD safely on the bedside, considering their unfavorable conditions for transfer to an operating room. Under sterile conditions with local anesthesia, a 20-gauge guide needle was inserted at the counter-McBurney point (the point over the left side of the abdomen that is one-third of the distance from the anterior superior iliac spine to the umbilicus) to inject 3–5 mL of sterile saline. We checked the insertion site of the catheter using abdominal sonography prior to puncture. As soon as the guide needle was inserted, we assessed the contents of the syringe to check for the presence of bowel injury. Thereafter, we inserted an intravenous (IV) cannula or a commercially available PD catheter with a guide wire for peritoneal access on the puncture site. The four types of catheters used were as follows: acute PD (APD) catheter (7.5, 8.5, and 9 Fr; Cook Inc., IN, USA), GamCath® catheter (8 Fr, dual lumens; Baxter, IL, USA), ARROW® central venous catheter (CVC) (4 Fr, dual lumens; Teleflex, NC, USA), and venous umbilical catheter (4 Fr; Vygon, Paris, France). Two to three side holes were manually created for the two types of vascular catheters, except for the APD catheter, during the procedure preparation. We evaluated the location of the catheter tip and determined the presence of other complications, including bowel perforation, using simple X-ray. The PD catheter tip was placed in the lower portion of the pelvis. Thereafter, PD was started immediately after the insertion of the catheter.

Dialysis solutions (Hemosol or Physioneal; Baxter International, Deerfield, IL, USA) were used at standard hydrous dextrose concentrations of 2.5 and 4.25% with osmolalities of 396 and 485 mOsm/L, respectively. To optimize ultrafiltration, the concentration of the dextrose in the dialysate was increased sequentially from 2.5 to 4.25% while monitoring the blood sugar level. PD was started at a rate of 10 mL/kg, which was increased to 20–30 mL/kg at 60–120 min/cycle continuing for 24 h; e.g. starting with input time, 20-min dwell time, 10-min outflow, 30 min in case of 60 min). At this point, since PD may cause hypotension in infants, the dwell time was set at a drainage period of 10 min. Whenever the blood pressure was not a problem in maintaining proper ultrafiltration and clearance, the dwell and outflow time were changed according to the levels of creatinine and electrolytes, metabolic status, and severity of edema. The initial PD cycle yielded an exchange inflow of 10 mL/kg for 10 min, a dwell time of 30 min, and a drain time of 20 min. We adjusted the dwell time and dextrose concentration of the dialysis fluids according to ultrafiltration (UF). UF refers to the difference between the osmotically induced ultrafiltration into the peritoneal cavity and the fluid loss from the cavity during dialysis [16]. UF was defined as a difference (mL) between the applied dialysis fluid and the amount of dialysate, and net UF (mL/kg/h) was calculated using body weight and the dialysis time. The amount of fluid that could be removed can be increased by increasing either the PD dextrose concentration, amount of fluid in the peritoneum, or frequency of exchanges. For input-dwell-output flow of the dialysis fluid, the ports of the three-way cannula connected to the PD catheter of the patients were manually operated by nurses at the appointed time. Gravity allows the filling and draining processes. Automated PD has not been available for ELBW infants because the minimum loading volume that supports the machine could not be satisfied [17]. To discontinue PD, we determined the weaning time based on increasing urine output, hemodynamic stability, and volume status.

### Clinical data of the neonates

We assessed the cause of AKI; indication and timing of PD; duration of PD (days); blood urea nitrogen (BUN), Cr, sodium, and potassium levels at pre- and post-PD; urine output (mL/kg/h) at pre- and post-PD; complications during PD; comorbidity; and causes of mortality. We also evaluated the use of nephrotoxic antibiotics (e.g., vancomycin, aminoglycoside, meropenem, and antifungal drugs), inotropes, and vasopressors before AKI diagnosis. Demographic characteristics, such as gestational age; birth weight; sex; history of multiple births; incidence rate of maternal preeclampsia; type of delivery; antenatal steroid use; Apgar scores at 1 and 5 min; presence of major morbidities, such as respiratory distress syndrome, patent ductus arteriosus (PDA), bronchopulmonary dysplasia (BPD) [18], significant neurological injury (SNI), necrotizing enterocolitis (NEC) [19], and late-onset sepsis (LOS); duration of hospital stay; and mortality rate, were also assessed for each subject. Significant PDA was defined as a condition requiring pharmacological and/or surgical treatment for the mitigation of hemodynamic disturbance. SNI included intraventricular hemorrhage (IVH) with grade ≥ 3 or periventricular leukomalacia. LOS was defined as bacterial infection confirmed by a positive blood or cerebrospinal fluid culture finding after postnatal day 3.

### Statistical analysis

All statistical analyses were performed using the SPSS version 20.0 software. To compare the multiple characteristics between AKI infants with and without PD, the independent t-and Mann-Whitney U tests were used for continuous variables. Chi-square and Fisher’s exact tests were used for categorical variables. *P* values < 0.05 were considered statistically significant.

## Results

**Fig. 1:**
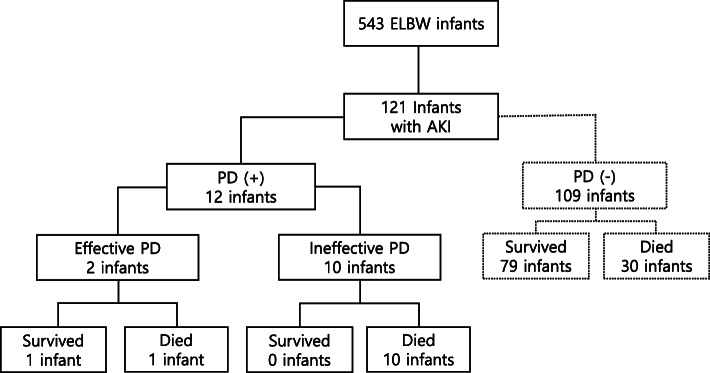
Prognosis of 543 ELBW infants. Effective dialysis is defined as the same or greater amount of drainage fluid volume than osmotic ultrafiltration. *Abbreviations:* ELBW, extremely low birth weight

Of 543 ELBW infants, 121 infants (22.3%) were diagnosed with AKI (Fig. 1). Twelve (seven male and five female infants) out of 121 infants were treated with PD. For AKI infants with PD, their mean gestational ages and birth weights were 27.2 (± 3.3) weeks and 706.5 (± 220.5) grams, respectively. All infants had no congenital defects of the genitourinary system. The demographic and clinical characteristics of AKI infants with PD and without PD were compared. There were no significant differences in demographics, such as gestational age, birth weight, and sex, and the Apgar score assigned immediately after birth between the groups. Intrauterine growth retardation was more frequent in AKI infants with PD. Mortality was also higher in the PD group. Further, there was no difference in morbidities such as PDA, BPD, SNI, NEC, ROP, and LOS between the groups (Table 1).

**Table 1:** Demographic and clinical characteristics of the enrolled subjects

	AKI infants with PD (*n* = 12)	AKI infants without PD (*n* = 109)	*P* Value
Gestational age (week), mean ± SD	27.2 ± 3.3	25.7 ± 2.1	0.080
Birth weight (g), mean ± SD	706.5 ± 220.5	681.4 ± 167.3	0.712
Male sex, *n* (%)	7 (58.3)	59 (54)	0.781
Apgar score at 1 min, median (range)	3 (0–7)	3 (0–7)	0.326
Apgar score at 5 min, median (range)	5 (1–8)	6 (1–9)	0.482
IUGR, *n* (%)	9 (75)	20 (18.4)	< 0.001
RDS, *n* (%)	11 (91.7)	106 (97.3)	0.345
Significant PDA, *n* (%)	6 (50)	46 (42.2)	0.605
Moderate to severe BPD, *n* (%)	5 (41.7)	48 (44)	0.875
Severe neurologic injury (IVH grade ≥ 3 or PVL), *n* (%)	8 (66.7)	39 (36)	0.058
NEC (stage ≥2), *n* (%)	0 (0)	21 (19.3)	0.124
ROP (stage ≥2), *n* (%)	2 (16.7)	32 (29.4)	0.506
LOS, *n* (%)	4 (33.3)	36 (33)	1.000
Hospital stay duration (day), mean ± SD	56.1 ± 49.1	81.5 ± 62.7	0.156
Mortality, *n* (%)	11 (91.7)	37 (34)	< 0.001

*SD* Standard deviation, *PROM* Premature rupture of membranes, *IUGR* Intrauterine growth retardation, *RDS* Respiratory distress syndrome, *PDA* Patent ductus arteriosus, *BPD* Bronchopulmonary dysplasia, *IVH* Intraventricular hemorrhage, *PVL* Periventricular leukomalacia, *NEC* Necrotizing enterocolitis, *ROP*, Retinopathy of prematurity, *LOS* Late-onset sepsis

Nine patients were classified as stage 3 according to neonatal KDIGO AKI classification, while the others were classified as stage 2. The most important cause of AKI was sepsis (50%) (Table 2). Two of the patients had AKI due to multiple organ dysfunction syndrome (MODS) after asphyxia in the delivery room. Two other patients started treatment with PD for bilateral renal vein thrombosis. Ten of the enrolled patients were administered with inotropes (e.g., dopamine, dobutamine, or epinephrine) and antibiotics (e.g., vancomycin, gentamicin, or meropenem) before the diagnosis of AKI (data not shown).

**Table 2:** Characteristics of peritoneal dialysis in the 12 ELBW infants with AKI

Patients	Weaning	GA (weeks)	Bwt (g)	Causes of AKI	Onset (days)	Type of catheters	Dwell time (minutes)	UF rate (mL/kg/h)	BUN	Cr	Sodium	Potassium	Urine output	Complications	Comorbidity	Outcome (Causes of mortality)
									pre−/post-PD (mg/dL)	pre−/ post-PD (mg/dL)	pre−/post-PD (mg/dL)	pre−/post-PD (mg/dL)	pre−/post-PD (mL/kg/h)			
1	NA	25 + 1	960	Sepsis	8	PD catheter (9 Fr)	80 → 30	0.72	74/65	4.73/3.15	147/140	5.7/3	0.1/0	Dialysate leakage, intraperitoneal hemorrhage, catheter obstruction	TTTS recipient	Mortality (Sepsis; MODS)
2	Y	26 + 4	990	MODS	3	UV catheter (4 Fr)	40	−0.06	24/70	1.76/2.35	124/165	1.76/4.8	0.25/0.26	Peritonitis, intraperitoneal hemorrhage		Mortality (MODS)
3	NA	25 + 2	420	Sepsis	43	ARROW catheter (4 Fr)	20	6.64	83/55	2.19/1.97	134/145	10.3/8	0.25/0.1			Mortality (Hyperkalemia)
4	NA	24 + 2	540	Sepsis	26	ARROW catheter (4 Fr) → PD catheter (8.5 Fr)	35	1.35	64/68	1.99/2.68	133/135	5.9/9.5	0.44/0	Dialysate leakage, catheter obstruction		Mortality (Hyperkalemia)
5	NA	26 + 6	760	Sepsis	43	PD catheter (8.5 Fr)	30	3.96	76/27	3/2.7	125/143	4.5/2.9	0/0	Dialysate leakage		Mortality (Sepsis; MODS, IVH)
6	NA	31 + 6	900	MODS	1	PD catheter (8.5 Fr)	30	0.84	27/10	1/1.2	138/148	3.9/3.3	0.18/0		Fetal hydrops	Mortality (Metabolic acidosis)
7	NA	35 + 5	960	Sepsis	20	PD catheter (8.5 Fr)	40 → 30	4.97	106/46	2.47/1.28	130/146	6.5/7	0/0		CoA	Mortality (Sepsis; MODS)
8	NA	26 + 6	550	Pulmonary hemorrhage	3	ARROW catheter (4.0 Fr)	45	6.54	39/25	2.7/2.5	134/153	8.1/4.5	1.2/0.23	Dialysate leakage	TTTS donor	Mortality (Pulmonary hemorrhage)
9	NA	23 + 6	470	Bilateral renal vein thrombosis	129	PD catheter (8.5 Fr)	30	3.46	94/70	2.8/2.6	131/140	7.2/5.7	0/0.2	Dialysate leakage		Mortality (Hyperkalemia)
10	N	26 + 3	868	Bilateral renal vein thrombosis	134	PD catheter (8.5 Fr)	30 → 60	2.61	98/68	2.6/2.2	150/138	7/2.5	0.61/0.1	Dialysate leakage, intraperitoneal hemorrhage	PA with IVS	Mortality (Hypotensive shock; MODS)
11	NA	27 + 6	590	Cardiogenic shock	3	PD catheter (8.5 Fr)	40	1.9	29/17	2.1/2.2	132/141	7.3/3.4	0.1/1.0		TTTS donor	Mortality (IVH, coma)
12	Y	27 + 3	470	Sepsis	13	PD catheter (7.5 Fr)	70	−0.12	66/26	2.92/0.88	151/142	9.7/6	1.13/5.7	Catheter obstruction	–	Survival

*ELBW* Extremely-low-birth-weight, *GA* Gestational age, *Bwt* Body weight, *AKI* Acute kidney injury, *PD* Peritoneal dialysis, *Fr* French, *UF* Ultrafiltration, *BUN* Blood urea nitrogen, *Cr* Creatinine, *MODS* Multiple organ dysfunction syndrome, *NA* Not available, *Y* Yes, *TTTS* Twin-to-twin transfusion syndrome, *CoA* Coarctation of aorta, *PA* Pulmonary atresia, *IVS* Intact ventricular septum, *IVH* Intraventricular hemorrhage

**Fig. 2:**
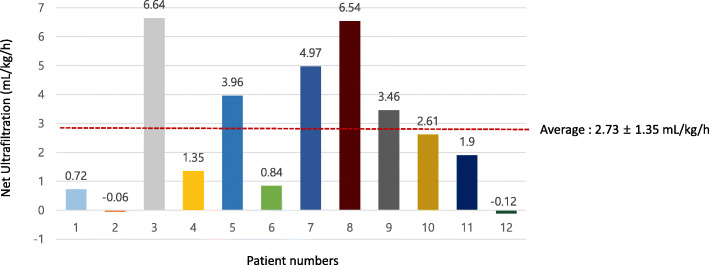
Net UF. The mean (±SD) UF rate was 2.73 (±1.35) mL/kg/h. *Abbreviations:* SD, standard deviation; UF, ultrafiltration

The mean age at the start of PD was 16.3 (± 16.3) days (range, 1–43 days), except for two patients who were more than 3 months old. If the two patients were included, the mean age was 35.5 (± 26.7) days (range, 1–134 days). The average duration of PD was 9.4 (±7.7) days (range, 5–14 days). The average percentage of weight gain before PD initiation was 14.4% (range, 2–53%). Treatment with PD fluids (2.5 or 4.25%) was started at a rate of 10 mL/kg, which was increased to 20–30 mL/kg at 60–120 min/cycle continuing for 24 h based on the effect of the dialysis. The concentration of PD fluid was increased from 2.5 to 4.25% in four patients owing to insufficient drainage. The concentration was decreased from 4.25 to 2.5% in only one patient owing to hyperglycemia. The average UF rate was 2.73 (±1.35) mL/kg/h (Fig. 2).

**Fig. 3:**
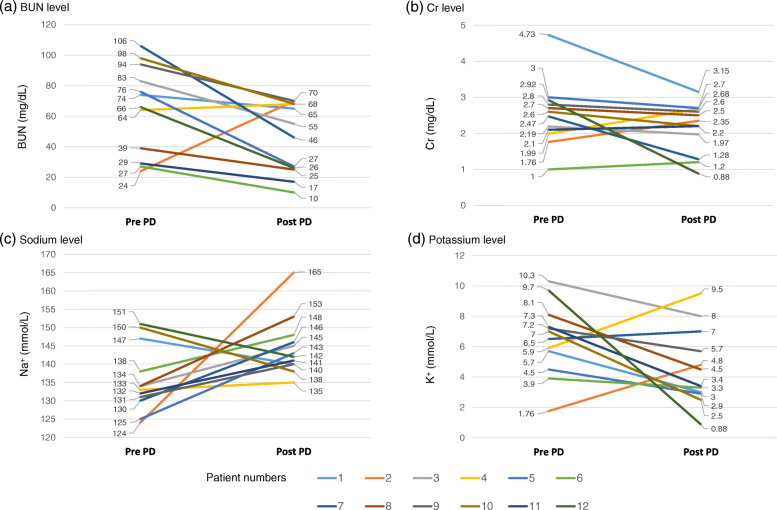
The serum BUN, Cr, sodium, and potassium levels before and after PD. The mean rate of reduction in the serum BUN and Cr levels during dialysis was 42.5% (range, 12–64%) and 20.1% (range, 0–70%), respectively (**a**, **b**). The sodium levels increased from 135.8 to 144.7 mg/mL (**c**), and the potassium levels decreased from 6.8 to 5.0 mg/mL after 9.3 (±4.4) days (**d**). *Abbreviations:* BUN, blood urea nitrogen; Cr, creatinine

To measure the effectiveness of the dialysis, the serum BUN, Cr, sodium, and potassium levels at pre- and post-PD were analyzed in 10 patients (Fig. 3). The two remaining patients died soon before the evaluation. The biochemical values recovered rapidly with PD. It was found that ELBW infants had wider peritoneal surface areas compared to their body weights. The average rate of reduction in the serum BUN and Cr levels during dialysis was 42.5% (range, 12–64%) and 20.1% (range, 0–70%), respectively. The sodium levels increased from 135.8 to 144.7 mg/mL, and the potassium levels decreased from 6.8 to 5.0 mg/mL after 9.3 (±4.4) days.

Complications related to PD were observed in eight patients (67%). The main complications were mechanical dysfunction of the catheter, including leakage at the insertion site and catheter obstruction. The most common mechanical dysfunction was dialysate leakage at the insertion site (75%). In this study, PD was discontinued in two patients possibly because of the low leakage of the PD catheter, which affected the net UF owing to the efficient PD action. Intraperitoneal hemorrhage at insertion or withdrawal of the catheter also occurred in three patients. Peritonitis was a severe complication of PD; it occurred in only one patient in this study. Peritonitis needing intraperitoneal antibiotic administration was the only infectious complication recorded.

Despite dialysis, four patients died owing to AKI-related hyperkalemia or metabolic acidosis. As soon as dialysis was performed, five other patients died owing to MODS caused by sepsis, respiratory arrest, and congestive heart failure. One patient died owing to pulmonary hemorrhage and another patient owing to deterioration of IVH regardless of dialysis.

Of the 12 patients, two patients were successfully weaned off dialysis after their renal function improved with negative net UF. One of them died owing to hypoxic encephalopathy and coma 28 days after withdrawal of PD. The only survivor completely recovered his renal function as defined by normalization of the serum BUN, Cr, and electrolyte levels; thus, he did not require long-term RRT. The mortality rate of the ELBW infants treated with PD was quite high (91.7%).

## Discussion

In NICUs, the use of dialysis in premature infants who are susceptible to AKI owing to their immaturity or exposure to infection and toxins is gradually increasing [4, 5, 17]. However, to date, there is no guideline-based evidence regarding the indications and methods of RRT for premature infants. If renal failure has not been resolved despite medical treatments, the timing and method of dialysis are selected on the basis of the decision of the primary care physician [8, 20, 21]. As a result, the prognosis of premature infants with AKI treated with RRT is expected to change. Especially, ELBW infants are at very high risk for AKI, but few studies have suggested criteria for successful dialysis in ELBW infants with AKI. Although a recent study reported the experience of PD in 3 ELBW infants, all infants were applied different types of the peritoneal catheters and technical methods without same guideline [14]. Therefore, despite dialysis, their mortality is quite high because patients’ organs are immature and, therefore, often have other organ failures [22].

Although infrequently reported [23], hemodialysis (HD) still may not be the treatment of choice in neonatal overdoses for the following reasons: (1) extracorporeal blood volumes for hemoperfusion are relatively large and maintenance of the circulatory dynamics in premature infants is challenging; (2) hemoperfusion filters require heparin to maintain patency; and (3) thrombocytopenia is a common complication of hemoperfusion. These factors can increase the risk of intracranial hemorrhage in neonates [20, 24]. Especially in ELBW infants, who have unstable blood pressure and in whom obtaining vascular access is difficult, HD can be barely applied effectively. Alternatively, continuous renal replacement therapy (CRRT) is also challenging in severely ill neonates where PD may not be suitable such as for patients with previous abdominal surgery. There are some limitations of CRRT in neonates. These include vascular access, bleeding complications, and lack of neonate-specific devices. An adequately-sized central venous access is needed to accommodate adequate blood flow rates and a CRRT machine meant for neonates should be adapted [25, 26]. A few studies have reported the use of CRRT in the NICU [25–27]. Although CRRT practices can be modified to fit the needs of neonates including preterms, there is a need for a device designed specifically for this population [25–27].

Over the years, PD has become an effective and increasingly popular alternative to HD in the management of critically ill neonates, including premature infants [9, 28]. PD has been relatively safe, technically simple, and cost-effective. It can also be applied in hemodynamically unstable premature infants [2, 8, 12, 28]. The catheter design, implantation site, and system configuration used to perform dialysis determine the effectiveness of PD in premature infants. However, the most common difficulty in PD is the introduction of a suitable peritoneal catheter for these patients. Obtaining catheter access for PD is more difficult in ELBW infants than in older neonates because of their small size and inelastic abdominal wall.

Permanent PD catheters (e.g., Tenckhoff catheter) with cuffs are very rigid and long for the small intra-abdominal cavities of infants. They need to be tunneled under the skin. However, for ELBW infants, PD catheters are inserted directly through the abdominal wall, without tunneling. Therefore, the insertion of permanent PD catheters among them is not easy. Conversely, temporary PD catheters are generally inserted along with IV catheters or commercially available peritoneal catheters [22]. Other alternatives are feeding tubes, suction catheters, neonatal chest drains, and Foley catheters [29–31]. In our study, although PD catheters were inserted to 8 of the 12 patients, IV catheters (e.g., ARROW® CVC and venous umbilical catheter) were inserted initially in another four patients owing to their low body weight. When IV catheters were used, we manually created some side holes during the procedure. We expected these holes to have better permeability to the membrane. Although these holes could have rough edges which may cause bowel perforation or intraperitoneal hemorrhage, no such complications were observed in our population. PD worked effectively in two ELBW infants with smaller-sized catheters. There was no difference in the risks of leakage, hemorrhage, and peritonitis according to the type of catheters.

Some complications associated with PD in premature infants include mechanical dysfunctions, such as dialysate leakage and catheter obstruction requiring revision or reinsertion, intraperitoneal hemorrhage, and bowel perforation [17, 20, 32]. Peritoneal fluid leakage around the PD catheter and along the tunnel is a serious problem that can increase the risk of bacterial and fungal peritonitis [21, 33]. In this study, the complications observed in relation to PD were mainly caused by catheter-related dialysate leakage, which was resolved after adjustment of the dwell volume or reinsertion of the catheter on the other side. In a previous study, a tissue adhesive, i.e., commercially available fibrin glue, was used at the insertion site [32]. Therefore, selection of an optimal catheter for PD is very important to minimize the complications associated with PD access. For peritonitis, the use of prophylactic antibiotics should be carefully considered with advantages of infection prevention and disadvantages of antibiotic-resistant bacterial generation [28, 31]. The efficacy of PD in ELBW infants is affected by many factors. In ELBW infants with hypotension, peripheral perfusion is insufficient for adequate exchange. Otherwise, if they develop sepsis, which increases vessel permeability, rapid solute removal and UF capacity reduction with gradient loss may occur [34].

Increasing the number of exchanges, administration of large volumes of dialysate, or adjustment of the concentration of glucose in dialysis fluids may be helpful in improving dialysis efficiency [35]. Although the number of exchanges may vary, approximately 24 exchanges per day are employed for PD. The number of exchanges is determined by the amount of fluid and solute removal required. A total of 20–40 cycles can be used; further, the procedure can be continued until the desired effect is obtained [35, 36]. The size of the peritoneal cavity, weight of the infant, presence of pulmonary or other diseases, and degree of uremic toxicity may influence the exchange volume [12, 37]. Additionally, it is rational to initiate PD using 2.5% dialysate solution to achieve better UF [12, 37]. Estimation of the peritoneal equilibration rate is necessary for optimal dialysis; however, practically frequent blood and dialysis fluid samplings are risky for ELBW infants. In our study, PD was started at a rate of 10 mL/kg, which was increased to 20–30 mL/kg at 60–120 min/cycle continuing for 24 h.

In this study, we emphasized the major technical challenges and lack of appropriate devices to perform PD in ELBW infants with AKI. As a result, the mortality rate of the ELBW infants treated with PD was quite high (91.7%) in this study. In addition, most of the patients had findings compatible with disseminated intravascular coagulation features. In a previous study, the mortality rate was 79% in ELBW infants treated with PD; they were assumed to have died owing to underlying medical conditions and multi-organ failure rather than renal failure [20, 28]. Herein, five ELBW infants had accompanying congenital heart disease and twin-to-twin transfusion. There were concerns on negative renal recovery; initiation of dialysis may decrease the urine output and intravascular volume. These could aggravate underlying diseases, including congenital heart disease, which would consequently affect the mortality rate of ELBW infants. However, the strength of the study is that it provides data on the effect of dialysis in ELBW infants with congenital heart disease or twin-to-twin transfusion; this effect has previously been monitored in only a few studies.

In this study, the main issue was whether this population should be resuscitated. It can be very difficult to decide whether to resuscitate ELBW infants with AKI who are less likely to survive. Since these infants may face various disabilities, chronic illnesses, multiple surgeries, and severe care dependency, deciding whether to resuscitate is both a clinical and an ethical decision [38]. A recent study has reported the degree of medical uncertainty and the fact that parents will deal with the consequences of decision making, highlighting the importance of providing a wide range of discretion in parental decision-making authority [39]. However, neonatologists are unable to determine the individual prognosis at birth and resuscitation has to be provided quickly for it to be successful. We continue to face great opportunities disguised as insoluble problems. Although the patients in this study had quite a high mortality rate, we expect our medical challenges to produce better prognosis in this population as the limit of viability gradually decreases. Defining these limits is important for developing local guidelines that will provide standards of practice to improve the morbidity and mortality of ELBW infants and for counseling parents [39]. Therefore, we hope that our study would be useful for developing local guidelines to both support health care practitioners and provide consistency of care for extremely preterm infants.

Recent studies have shown that acute PD is still an appropriate treatment option for VLBW newborns with AKI [15, 32, 40]. Furthermore, some studies have reported PD experiences in extremely immature infants [14, 17, 20–23, 31, 40, 41]. Although this study has an increased mortality rate for ELBW infants who underwent PD, these technical challenges can be overcome. Improvised PD systems and catheters in ELBW infants may produce results comparable to those of VLBW infants. The outcomes of ELBW infants with AKI would then be greatly improved.

Our study also has some limitations. First, it was a retrospective study of a relatively small number of infants conducted at a single center; thus, the findings might not extrapolate to a larger population. Second, our data do not provide evidence regarding whether nutritional intake of ELBW infants causes high morbidity and mortality rates in maintenance dialysis. Lastly, this study did not include premature infants with contraindications of PD, such as NEC, severe respiratory failure, and hemorrhagic tendency. The intrusion of the peritoneal cavity or placement of multiple abdominal drains can be attributed to these contraindications. Given the available data on the comparison between ELBW infants with and without PD for AKI, we should consider the technical difficulties of PD and alternatives to HD for the treatment of premature infants.

## Conclusions

Considering that no data regarding the validity and efficacy of hemodialysis are currently available, PD can be considered the only rescue therapy for ELBW infants with acute renal failure. PD in ELBW infants seems to be a feasible procedure without major complications. However, ethically speaking, the use of PD should be carefully considered when treating ELBW infants with AKI because of the high mortality. Forward, it is necessary to increase guideline-based evidence regarding the indications and methods of PD for ELBW infants with AKI, which can apply PD at the appropriate time, and reduce their complications and mortality rate related to PD.

## Data Availability

The datasets used and/or analyzed in the current study are available from the corresponding author on reasonable request.

## Abbreviations

AKI: Acute kidney injury
PD: Peritoneal dialysis
ELBW: Extremely-low-birth-weight
NICU: Neonatal intensive care unit
RRT: Renal replacement therapy
APD: Acute peritoneal dialysis
Cr: Creatinine
IV: Intravenous
CVC: Central venous catheter
UF: Ultrafiltration
BUN: Blood urea nitrogen
PDA: Patent ductus arteriosus
BPD: Bronchopulmonary dysplasia
SNI: Significant neurological injury
NEC: Necrotizing enterocolitis
LOS: Late-onset sepsis
IVH: Intraventricular hemorrhage
MODS: Multiple organ dysfunction syndrome
HD: Hemodialysis
